# Supplementary material for: PET and CSF amyloid-β status are differently predicted by patient features: information from discordant cases
Source: Alzheimers Res Ther. 2019 Dec 7;11:100. doi: 10.1186/s13195-019-0561-5 (PMC6898919; doi:10.1186/s13195-019-0561-5)
Supplement: Supplementary file 1 — Additional file 1: Table S1. Proportion of missing values per patient feature. Table S2. Out-of-bag accuracy, sensitivity and specificity for random forest models predicting amyloid PET and CSF status. Figure S1. Relative predictive power of patient features for amyloid PET and CSF status when removing PET-CSF- MCI and dementia patients. Table S3. Predictive value of patient features for amyloid status based on PET or CSF. Table S4. Amyloid-adjusted predictive value of patient features for amyloid status based on PET or CSF. [file 13195_2019_561_MOESM1_ESM.docx]

|  | **PET-CSF-** | **PET+CSF-** | **PET-CSF+** | **PET+CSF+** |
| --- | --- | --- | --- | --- |
| n | 315 | 32 | 65 | 356 |
| Age (%) | 0 (0.0) | 0 (0.0) | 0 (0.0) | 0 (0.0) |
| Sex (%) | 0 (0.0) | 0 (0.0) | 0 (0.0) | 0 (0.0) |
| Education (%) | 13 (4.1) | 2 (6.2) | 3 (4.6) | 10 (2.8) |
| APOE ε4 (%) | 12 (3.8) | 2 (6.2) | 4 (6.2) | 14 (3.9) |
| CSF tau (%) | 0 (0.0) | 0 (0.0) | 1 (1.5) | 2 (0.6) |
| CSF p-tau (%) | 2 (0.6) | 0 (0.0) | 4 (6.2) | 0 (0.0) |
| MMSE (%) | 5 (1.6) | 1 (3.1) | 2 (3.1) | 7 (2.0) |
| Memory z-score (%) | 14 (4.4) | 3 (9.4) | 3 (4.6) | 21 (5.9) |
| Language z-score (%) | 17 (5.4) | 3 (9.4) | 3 (4.6) | 25 (7.0) |
| Attention z-score (%) | 15 (4.8) | 3 (9.4) | 3 (4.6) | 22 (6.2) |
| Executive z-score (%) | 6 (1.9) | 1 (3.1) | 2 (3.1) | 12 (3.4) |
| Visuospatial z-score (%) | 23 (7.3) | 3 (9.4) | 5 (7.7) | 36 (10.1) |
| MRI MTA (%) | 68 (21.6) | 4 (12.5) | 6 (9.2) | 88 (24.7) |
| MRI PCA (%) | 91 (28.9) | 4 (12.5) | 7 (10.8) | 93 (26.1) |
| MRI Fazekas (%) | 68 (21.6) | 4 (12.5) | 2 (3.1) | 89 (25.0) |
| MRI lacunes (%) | 76 (24.1) | 5 (15.6) | 3 (4.6) | 95 (26.7) |
| MRI microbleeds (%) | 78 (24.8) | 5 (15.6) | 10 (15.4) | 102 (28.7) |

**Supplementary Table 1**. Proportion of missing values per patient feature

|  | **Outcome** | **Accuracy %** | **Sensitivity %** | **Specificity %** |
| --- | --- | --- | --- | --- |
|  |  |  |  |  |
| **Total** | PET | 82 (81, 83) | 82 (81, 83) | 82 (81, 83) |
|  | CSF | 78 (77, 78) | 81 (80, 82) | 74 (73, 75) |
| **SCD** | PET | 82 (80, 82) | 22 (18, 26) | 96 (96, 96) |
|  | CSF | 79 (77, 80) | 31 (27, 36) | 94 (94, 95) |
| **MCI** | PET | 82 (80, 84) | 81 (79, 83) | 84 (82, 84) |
|  | CSF | 72 (69, 75) | 74 (70, 77) | 70 (65, 75) |
| **Dementia** | PET | 82 (82, 83) | 90 (89, 91) | 69 (68, 71) |
|  | CSF | 78 (77, 80) | 90 (89, 91) | 53 (50, 57) |

**Supplementary Table 2**. Out-of-bag accuracy, sensitivity and specificity for random forest models predicting amyloid PET and CSF status

Mean rates with 95% confidence intervals over 25 random forest models are reported.

**Supplementary Figure 1**. Relative predictive power of patient features for amyloid PET and CSF status when removing PET-CSF- MCI and dementia patients


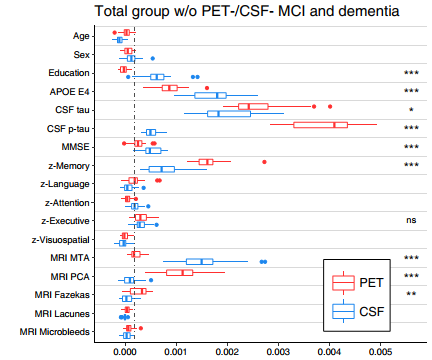


AUC-based variable importance (VIM) from 25 random forest models predicting PET status and 25 models from predicting CSF status are plotted. P-values (*** - p<0.001, ** - p<0.01, * - p<0.05, ns - non-significant) indicate the bootstrapped difference of VIM values between models predicting PET and CSF status.

|  |  | **TOTAL** | | | | | **SCD** | | | | | | **MCI** | | | | | | **DEMENTIA** | | | | |
| --- | --- | --- | --- | --- | --- | --- | --- | --- | --- | --- | --- | --- | --- | --- | --- | --- | --- | --- | --- | --- | --- | --- | --- |
|  |  |  |  |  | Imputed | |  |  |  | Imputed | |  | |  |  | Imputed | |  | |  |  | Imputed | |
| **Predictor** | **Out-**  **come** | **Odds ratio**  **(95% CI)** | **p unc** | **p FDR** | **Odds ratio**  **(95% CI)** | **p FDR** | **Odds ratio**  **(95% CI)** | **p unc** | **p FDR** | **Odds ratio**  **(95% CI)** | **p FDR** | **Odds ratio**  **(95% CI)** | | **p unc** | **p FDR** | **Odds ratio**  **(95% CI)** | **p FDR** | **Odds ratio**  **(95% CI)** | | **p unc** | **p FDR** | **Odds ratio**  **(95% CI)** | **p FDR** |
| Age | PET | 1.02(1.00,1.04) |  |  | 1.02(1.00,1.04) |  | 1.06(1.01,1.12) | * |  | 1.06(1.01,1.12) |  | 0.96(0.92,1.00) | |  |  | 0.96(0.92,1.00) |  | 1.00(0.97,1.03) | |  |  | 1.00(0.97,1.03) |  |
|  | CSF | 1.01(0.99,1.03) |  |  | 1.01(0.99,1.03) |  | 1.04(1.00,1.09) |  |  | 1.04(1.00,1.09) |  | 0.96(0.92,1.00) | |  |  | 0.96(0.92,1.00) |  | 0.98(0.96,1.01) | |  |  | 0.98(0.96,1.01) |  |
| Sex, F | PET | 1.69(1.26,2.26) | *** | *** | 1.69(1.26,2.26) | *** | 1.64(0.81,3.36) |  |  | 1.64(0.81,3.36) |  | 2.45(1.14,5.26) | | * |  | 2.45(1.14,5.26) |  | 1.70(1.13,2.53) | | ** | * | 1.70(1.13,2.53) | * |
|  | CSF | 1.55(1.15,2.07) | ** | ** | 1.55(1.15,2.07) | ** | 1.91(0.99,3.69) |  |  | 1.91(0.99,3.69) |  | 2.01(0.94,4.28) | |  |  | 2.01(0.94,4.28) |  | 1.40(0.93,2.12) | |  |  | 1.40(0.93,2.12) |  |
| Education | PET | 1.06(0.94,1.19) |  |  | 1.07(0.95,1.20) |  | 1.10(0.84,1.46) |  |  | 1.10(0.84,1.45) |  | 0.92(0.69,1.24) | |  |  | 0.91(0.68,1.22) |  | 1.28(1.08,1.52) | | ** | ** | 1.30(1.10,1.53) | ** |
|  | CSF | 1.04(0.93,1.17) |  |  | 1.05(0.94,1.18) |  | 1.05(0.82,1.35) |  |  | 1.04(0.81,1.34) |  | 0.93(0.69,1.24) | |  |  | 0.94(0.71,1.26) |  | 1.29(1.08,1.54) | | ** | * | 1.30(1.09,1.54) | ** |
| APOE E4 | PET | 4.72(3.46,6.44) | *** | *** | 4.57(3.34,6.24) | *** | 2.97(1.42,6.20) | ** | * | 2.97(1.42,6.19) | * | 14.55(6.08,34.82) | | *** | *** | 13.43(5.62,32.11) | *** | 3.63(2.39,5.50) | | *** | *** | 3.51(2.30,5.34) | *** |
|  | CSF | 4.60(3.36,6.28) | *** | *** | 4.46(3.26,6.12) | *** | 3.82(1.90,7.70) | *** | ** | 3.75(1.86,7.57) | ** | 8.28(3.70,18.54) | | *** | *** | 7.76(3.49,17.27) | *** | 3.68(2.39,5.69) | | *** | *** | 3.59(2.33,5.53) | *** |
| CSF tau | PET | 1.005  (1.004,1.006) | *** | *** | 1.005  (1.004,1.006) | *** | 1.004  (1.002,1.006) | *** | *** | 1.004  (1.002,1.006) | *** | 1.008  (1.005,1.011) | | *** | *** | 1.008  (1.005,1.011) | *** | 1.004  (1.003,1.005) | | *** | *** | 1.004  (1.003,1.005) | *** |
|  | CSF | 1.004  (1.003,1.005) | *** | *** | 1.004  (1.003,1.005) | *** | 1.003  (1.002,1.005) | *** | ** | 1.003  (1.002,1.005) | ** | 1.003  (1.002,1.005) | | *** | *** | 1.003  (1.002,1.005) | ** | 1.004  (1.003,1.004) | | *** | *** | 1.003  (1.002,1.004) | *** |
| CSF p-tau | PET | 1.05(1.04,1.06) | *** | *** | 1.05(1.04,1.06) | *** | 1.04(1.02,1.05) | *** | *** | 1.04(1.02,1.05) | *** | 1.05(1.03,1.07) | | *** | *** | 1.05(1.03,1.07) | *** | 1.05(1.04,1.06) | | *** | *** | 1.05(1.04,1.06) | *** |
|  | CSF | 1.04(1.03,1.04) | *** | *** | 1.04(1.03,1.04) | *** | 1.03(1.01,1.04) | *** | ** | 1.02(1.01,1.04) | ** | 1.03(1.01,1.04) | | *** | *** | 1.03(1.01,1.04) | ** | 1.04(1.03,1.05) | | *** | *** | 1.04(1.03,1.05) | *** |
| MMSE | PET | 1.20(1.15,1.25) | *** | *** | 1.19(1.14,1.24) | *** | 1.03(0.89,1.19) |  |  | 1.02(0.88,1.18) |  | 1.11(0.95,1.30) | |  |  | 1.12(0.95,1.31) |  | 1.12(1.06,1.18) | | *** | *** | 1.12(1.06,1.18) | *** |
|  | CSF | 1.20(1.15,1.25) | *** | *** | 1.19(1.14,1.25) | *** | 1.15(1.01,1.31) | * |  | 1.13(1.00,1.29) |  | 1.02(0.87,1.20) | |  |  | 1.02(0.87,1.19) |  | 1.10(1.04,1.17) | | *** | ** | 1.10(1.04,1.17) | ** |
| Memory | PET | 1.36(1.27,1.47) | *** | *** | 1.36(1.27,1.46) | *** | 1.13(0.82,1.55) |  |  | 1.14(0.83,1.56) |  | 1.26(1.02,1.57) | | * |  | 1.25(1.00,1.54) |  | 1.20(1.10,1.30) | | *** | *** | 1.20(1.10,1.31) | *** |
|  | CSF | 1.32(1.23,1.42) | *** | *** | 1.32(1.23,1.42) | *** | 1.23(0.92,1.64) |  |  | 1.22(0.91,1.62) |  | 1.16(0.95,1.42) | |  |  | 1.12(0.92,1.38) |  | 1.14(1.05,1.24) | | ** | ** | 1.15(1.06,1.26) | ** |
| Language | PET | 1.10(1.00,1.20) |  |  | 1.09(0.99,1.20) |  | 0.95(0.56,1.60) |  |  | 0.94(0.56,1.57) |  | 0.38(0.18,0.81) | | * |  | 0.44(0.21,0.95) |  | 0.94(0.85,1.04) | |  |  | 0.94(0.85,1.04) |  |
|  | CSF | 1.20(1.07,1.34) | ** | ** | 1.19(1.07,1.33) | ** | 0.99(0.62,1.58) |  |  | 0.99(0.62,1.56) |  | 0.71(0.39,1.27) | |  |  | 0.71(0.39,1.29) |  | 1.00(0.90,1.11) | |  |  | 1.00(0.90,1.11) |  |
| Attention | PET | 1.31(1.14,1.49) | *** | *** | 1.27(1.12,1.45) | *** | 1.06(0.72,1.55) |  |  | 1.00(0.69,1.46) |  | 0.57(0.34,0.94) | | * |  | 0.66(0.41,1.05) |  | 1.03(0.86,1.23) | |  |  | 1.01(0.85,1.19) |  |
|  | CSF | 1.36(1.19,1.56) | *** | *** | 1.32(1.16,1.50) | *** | 1.10(0.77,1.55) |  |  | 1.07(0.76,1.50) |  | 0.86(0.54,1.37) | |  |  | 0.98(0.62,1.53) |  | 1.00(0.83,1.20) | |  |  | 0.97(0.81,1.16) |  |
| Executive | PET | 1.28(1.15,1.42) | *** | *** | 1.28(1.15,1.42) | *** | 1.02(0.72,1.45) |  |  | 1.02(0.72,1.44) |  | 0.68(0.45,1.04) | |  |  | 0.70(0.46,1.05) |  | 0.95(0.82,1.11) | |  |  | 0.96(0.82,1.11) |  |
|  | CSF | 1.27(1.14,1.41) | *** | *** | 1.27(1.14,1.42) | *** | 1.05(0.76,1.44) |  |  | 1.05(0.76,1.44) |  | 0.82(0.55,1.22) | |  |  | 0.83(0.55,1.24) |  | 0.88(0.76,1.03) | |  |  | 0.89(0.76,1.04) |  |
| Visuo-  spatial | PET | 1.36(1.22,1.52) | *** | *** | 1.33(1.19,1.48) | *** | 0.92(0.59,1.45) |  |  | 0.89(0.56,1.41) |  | 0.72(0.47,1.08) | |  |  | 0.78(0.52,1.16) |  | 1.30(1.15,1.49) | | *** | *** | 1.25(1.10,1.43) | ** |
|  | CSF | 1.38(1.23,1.55) | *** | *** | 1.34(1.19,1.50) | *** | 1.34(0.93,1.93) |  |  | 1.35(0.95,1.93) |  | 0.98(0.68,1.42) | |  |  | 1.02(0.71,1.47) |  | 1.21(1.07,1.37) | | ** | ** | 1.16(1.03,1.31) | * |
| MRI MTA | PET | 1.27(1.05,1.53) | * | * | 1.25(1.04,1.50) | * | 1.78(0.90,3.53) |  |  | 1.56(0.80,3.04) |  | 0.77(0.48,1.24) | |  |  | 0.75(0.47,1.20) |  | 0.77(0.60,1.00) | |  |  | 0.81(0.63,1.04) |  |
|  | CSF | 1.40(1.16,1.70) | *** | ** | 1.34(1.11,1.61) | ** | 1.47(0.76,2.84) |  |  | 1.37(0.72,2.59) |  | 1.09(0.69,1.73) | |  |  | 0.98(0.62,1.54) |  | 0.81(0.62,1.05) | |  |  | 0.84(0.65,1.09) |  |
| MRI PCA | PET | 1.65(1.32,2.07) | *** | *** | 1.62(1.30,2.01) | *** | 1.71(0.93,3.16) |  |  | 1.55(0.87,2.78) |  | 0.84(0.47,1.51) | |  |  | 0.85(0.48,1.51) |  | 1.20(0.88,1.62) | |  |  | 1.18(0.87,1.59) |  |
|  | CSF | 1.38(1.11,1.73) | ** | ** | 1.41(1.14,1.74) | ** | 1.07(0.60,1.90) |  |  | 1.08(0.61,1.90) |  | 0.74(0.41,1.32) | |  |  | 0.75(0.42,1.32) |  | 0.97(0.70,1.34) | |  |  | 1.02(0.75,1.40) |  |
| MRI Fazekas | PET | 1.02(0.82,1.27) |  |  | 1.01(0.81,1.25) |  | 0.98(0.53,1.83) |  |  | 0.92(0.50,1.71) |  | 0.83(0.48,1.42) | |  |  | 0.78(0.45,1.38) |  | 0.80(0.60,1.06) | |  |  | 0.83(0.62,1.11) |  |
|  | CSF | 1.19(0.95,1.48) |  |  | 1.12(0.90,1.39) |  | 1.38(0.79,2.41) |  |  | 1.25(0.72,2.16) |  | 0.77(0.45,1.32) | |  |  | 0.74(0.42,1.30) |  | 0.96(0.71,1.31) | |  |  | 0.95(0.70,1.30) |  |
| MRI Lacunes | PET | 0.84(0.43,1.62) |  |  | 0.85(0.44,1.65) |  | 0(0,Inf) |  |  | 0(0,Inf) |  | 0.33(0.06,1.70) | |  |  | 0.36(0.07,1.83) |  | 0.72(0.33,1.59) | |  |  | 0.73(0.33,1.63) |  |
|  | CSF | 1.44(0.73,2.85) |  |  | 1.25(0.63,2.46) |  | 6.16(0.54,69.92) |  |  | 3.58(0.35,36.7) |  | 0.31(0.06,1.63) | |  |  | 0.37(0.07,2.00) |  | 1.25(0.51,3.05) | |  |  | 1.06(0.44,2.58) |  |
| MRI  microbleeds | PET | 1.91(1.21,3.01) | ** | ** | 1.59(1.01,2.50) |  | 2.17(0.75,6.30) |  |  | 1.84(0.68,4.99) |  | 1.32(0.52,3.32) | |  |  | 1.11(0.47,2.62) |  | 2.36(1.16,4.81) | | * | * | 1.93(0.93,3.99) |  |
|  | CSF | 1.51(0.96,2.39) |  |  | 1.35(0.86,2.10) |  | 1.49(0.52,4.25) |  |  | 1.32(0.49,3.52) |  | 1.06(0.42,2.65) | |  |  | 0.98(0.41,2.32) |  | 1.98(0.95,4.13) | |  |  | 1.70(0.81,3.56) |  |

**Supplementary Table 3.** Predictive value of patient features for amyloid status based on PET or CSF

*** - p<0.001, ** - p<0.01, * - p<0.05. P-values indicate the significance of the patient feature in the model. Uncorrected p-values and corrected p-values are reported per model, additionally corrected p-values for imputed data. False discovery rate (FDR) correction was performed for multiple comparisons. Cognitive scores have been multiplied by -1, therefore lower scores usually indicate higher odds ratios for amyloid positivity.

**Supplementary Table 4**. Amyloid-adjusted predictive value of patient features for amyloid status based on PET or CSF

|  |  | **TOTAL** | | | | | **SCD** | | | | | **MCI** | | | | | | **DEMENTIA** | | | | |
| --- | --- | --- | --- | --- | --- | --- | --- | --- | --- | --- | --- | --- | --- | --- | --- | --- | --- | --- | --- | --- | --- | --- |
|  |  |  |  |  | Imputed | |  |  |  | Imputed | |  |  |  | Imputed | |  | |  |  | Imputed | |
| **Predictor** | **Out-**  **come** | **Odds ratio**  **(95% CI)** | **p unc** | **p FDR** | **Odds ratio**  **(95% CI)** | **p FDR** | **Odds ratio**  **(95% CI)** | **p unc** | **p FDR** | **Odds ratio**  **(95% CI)** | **p FDR** | **Odds ratio**  **(95% CI)** | **p unc** | **p FDR** | **Odds ratio**  **(95% CI)** | **p FDR** | **Odds ratio**  **(95% CI)** | | **p unc** | **p FDR** | **Odds ratio**  **(95% CI)** | **p FDR** |
| Age | PET | 1.03(1.00,1.06) |  |  | 1.03(1.00,1.06) |  | 1.04(0.99,1.10) |  |  | 1.04(0.99,1.10) |  | 0.97(0.91,1.04) |  |  | 0.97(0.91,1.04) |  | 1.03(0.99,1.07) | |  |  | 1.03(0.99,1.07) |  |
|  | CSF | 0.99(0.96,1.02) |  |  | 0.99(0.96,1.02) |  | 1.02(0.97,1.07) |  |  | 1.02(0.97,1.07) |  | 0.98(0.92,1.04) |  |  | 0.98(0.92,1.04) |  | 0.96(0.93,1.00) | |  |  | 0.96(0.93,1.00) |  |
| Sex, F | PET | 1.57(1.01,2.44) | * |  | 1.57(1.01,2.44) |  | 1.17(0.49,2.77) |  |  | 1.17(0.49,2.77) |  | 2.27(0.75,6.90) |  |  | 2.27(0.75,6.90) |  | 1.93(1.04,3.58) | | * |  | 1.93(1.04,3.58) |  |
|  | CSF | 1.10(0.71,1.72) |  |  | 1.10(0.71,1.72) |  | 1.76(0.80,3.90) |  |  | 1.76(0.80,3.90) |  | 1.11(0.37,3.35) |  |  | 1.11(0.37,3.35) |  | 0.84(0.44,1.59) | |  |  | 0.84(0.44,1.59) |  |
| Education | PET | 1.06(0.89,1.27) |  |  | 1.07(0.89,1.27) |  | 1.11(0.79,1.56) |  |  | 1.12(0.79,1.57) |  | 0.95(0.60,1.50) |  |  | 0.90(0.57,1.41) |  | 1.16(0.90,1.49) | |  |  | 1.18(0.92,1.51) |  |
|  | CSF | 1.00(0.84,1.19) |  |  | 1.00(0.84,1.19) |  | 1.00(0.74,1.35) |  |  | 0.99(0.73,1.34) |  | 0.97(0.61,1.54) |  |  | 1.02(0.65,1.60) |  | 1.15(0.89,1.50) | |  |  | 1.14(0.88,1.48) |  |
| APOE E4 | PET | 2.58(1.65,4.03) | *** | *** | 2.52(1.62,3.93) | *** | 1.54(0.62,3.78) |  |  | 1.56(0.63,3.82) |  | 9.44(2.93,30.39) | *** | ** | 8.79(2.72,28.41) | ** | 2.22(1.20,4.09) | | * |  | 2.14(1.16,3.95) |  |
|  | CSF | 2.30(1.47,3.60) | *** | ** | 2.28(1.45,3.57) | ** | 3.07(1.33,7.07) | ** |  | 3.01(1.30,6.94) |  | 1.85(0.58,5.92) |  |  | 1.85(0.58,5.88) |  | 2.00(1.06,3.78) | | * |  | 2.00(1.07,3.75) |  |
| CSF tau | PET | 1.003  (1.003,1.004) | *** | *** | 1.003  (1.003,1.004) | *** | 1.003  (1.001,1.005) | ** | * | 1.003  (1.001,1.005) | * | 1.008  (1.004,1.012) | *** | ** | 1.008  (1.004,1.012) | ** | 1.003  (1.002,1.004) | | *** | *** | 1.003  (1.002,1.004) | *** |
|  | CSF | 1.001  (1.000,1.002) | ** | * | 1.001  (1.000,1.002) | * | 1.002  (1.000,1.003) |  |  | 1.001  (1.000,1.003) |  | 0.999  (0.997,1.001) |  |  | 0.999  (0.997,1.001) |  | 1.001  (1.000,1.002) | |  |  | 1.001  (1.000,1.002) |  |
| CSF p-tau | PET | 1.04(1.03,1.05) | *** | *** | 1.04(1.03,1.05) | *** | 1.02(1.01,1.04) | ** | * | 1.03(1.01,1.04) | * | 1.05(1.02,1.07) | *** | ** | 1.05(1.02,1.07) | ** | 1.04(1.03,1.05) | | *** | *** | 1.04(1.03,1.05) | *** |
|  | CSF | 1.01(1.00,1.02) | * |  | 1.01(1.00,1.02) |  | 1.01(1.00,1.03) |  |  | 1.01(0.99,1.02) |  | 0.99(0.98,1.01) |  |  | 0.99(0.98,1.01) |  | 1.01(1.00,1.02) | |  |  | 1.01(1.00,1.02) |  |
| MMSE | PET | 1.11(1.05,1.17) | *** | ** | 1.10(1.04,1.17) | ** | 0.93(0.80,1.10) |  |  | 0.93(0.79,1.09) |  | 1.22(0.96,1.56) |  |  | 1.24(0.97,1.59) |  | 1.10(1.02,1.19) | | * |  | 1.10(1.01,1.18) |  |
|  | CSF | 1.10(1.04,1.16) | ** | ** | 1.10(1.04,1.16) | ** | 1.21(1.03,1.41) | * |  | 1.19(1.02,1.38) |  | 0.88(0.69,1.12) |  |  | 0.87(0.69,1.11) |  | 1.02(0.94,1.10) | |  |  | 1.02(0.94,1.11) |  |
| Memory | PET | 1.22(1.12,1.34) | *** | *** | 1.22(1.12,1.33) | *** | 0.99(0.69,1.42) |  |  | 1.01(0.70,1.46) |  | 1.25(0.96,1.64) |  |  | 1.27(0.97,1.65) |  | 1.18(1.05,1.32) | | ** | * | 1.17(1.05,1.31) |  |
|  | CSF | 1.09(1.00,1.19) | * |  | 1.09(1.01,1.19) |  | 1.23(0.87,1.75) |  |  | 1.21(0.85,1.72) |  | 0.96(0.71,1.30) |  |  | 0.92(0.68,1.25) |  | 1.00(0.89,1.11) | |  |  | 1.01(0.91,1.12) |  |
| Language | PET | 0.95(0.85,1.07) |  |  | 0.95(0.84,1.07) |  | 0.91(0.45,1.86) |  |  | 0.91(0.46,1.80) |  | 0.23(0.08,0.68) | ** |  | 0.32(0.10,1.01) |  | 0.90(0.79,1.01) | |  |  | 0.89(0.79,1.01) |  |
|  | CSF | 1.24(1.08,1.43) | ** | * | 1.23(1.07,1.42) | * | 1.03(0.58,1.82) |  |  | 1.02(0.59,1.78) |  | 1.59(0.77,3.27) |  |  | 1.37(0.63,2.98) |  | 1.12(0.95,1.32) | |  |  | 1.13(0.96,1.34) |  |
| Attention | PET | 1.10(0.91,1.34) |  |  | 1.09(0.90,1.32) |  | 1.00(0.65,1.52) |  |  | 0.96(0.63,1.45) |  | 0.38(0.18,0.80) | * |  | 0.43(0.21,0.86) |  | 1.07(0.81,1.40) | |  |  | 1.07(0.82,1.39) |  |
|  | CSF | 1.27(1.03,1.55) | * |  | 1.24(1.02,1.50) |  | 1.10(0.71,1.70) |  |  | 1.09(0.72,1.66) |  | 1.80(0.88,3.68) |  |  | 1.87(0.93,3.77) |  | 0.95(0.72,1.26) | |  |  | 0.92(0.70,1.21) |  |
| Executive | PET | 1.17(1.00,1.37) |  |  | 1.16(1.00,1.36) |  | 0.99(0.67,1.48) |  |  | 0.99(0.67,1.47) |  | 0.61(0.33,1.12) |  |  | 0.62(0.34,1.14) |  | 1.11(0.88,1.40) | |  |  | 1.10(0.87,1.39) |  |
|  | CSF | 1.12(0.96,1.31) |  |  | 1.13(0.97,1.32) |  | 1.05(0.71,1.55) |  |  | 1.05(0.72,1.54) |  | 1.18(0.64,2.17) |  |  | 1.17(0.64,2.15) |  | 0.81(0.64,1.03) | |  |  | 0.82(0.64,1.04) |  |
| Visuo-  spatial | PET | 1.19(1.03,1.37) | * | * | 1.17(1.03,1.34) |  | 0.77(0.49,1.22) |  |  | 0.73(0.46,1.18) |  | 0.55(0.31,0.96) | * |  | 0.61(0.36,1.03) |  | 1.32(1.10,1.59) | | ** | * | 1.28(1.07,1.53) |  |
|  | CSF | 1.20(1.04,1.39) | * | * | 1.16(1.01,1.34) |  | 1.53(0.99,2.38) |  |  | 1.58(1.01,2.45) |  | 1.63(0.93,2.83) |  |  | 1.53(0.89,2.63) |  | 0.99(0.84,1.17) | |  |  | 0.97(0.83,1.13) |  |
| MRI MTA | PET | 1.00(0.76,1.31) |  |  | 1.02(0.78,1.33) |  | 1.68(0.77,3.68) |  |  | 1.48(0.66,3.32) |  | 0.55(0.28,1.05) |  |  | 0.58(0.30,1.09) |  | 0.79(0.55,1.15) | |  |  | 0.81(0.56,1.17) |  |
|  | CSF | 1.37(1.06,1.77) | * | * | 1.30(1.01,1.68) |  | 1.15(0.53,2.51) |  |  | 1.12(0.51,2.43) |  | 1.78(0.87,3.63) |  |  | 1.52(0.76,3.03) |  | 0.96(0.67,1.38) | |  |  | 0.99(0.69,1.41) |  |
| MRI PCA | PET | 1.71(1.25,2.33) | *** | ** | 1.66(1.22,2.26) | ** | 1.87(0.93,3.76) |  |  | 1.74(0.88,3.43) |  | 1.09(0.48,2.46) |  |  | 1.11(0.50,2.48) |  | 1.50(0.97,2.31) | |  |  | 1.42(0.93,2.17) |  |
|  | CSF | 0.95(0.70,1.29) |  |  | 0.96(0.72,1.30) |  | 0.78(0.39,1.56) |  |  | 0.78(0.39,1.57) |  | 0.69(0.30,1.58) |  |  | 0.69(0.31,1.52) |  | 0.74(0.47,1.14) | |  |  | 0.79(0.51,1.21) |  |
| MRI Fazekas | PET | 0.82(0.61,1.11) |  |  | 0.84(0.62,1.14) |  | 0.76(0.36,1.62) |  |  | 0.70(0.33,1.50) |  | 0.98(0.46,2.08) |  |  | 0.95(0.45,2.02) |  | 0.68(0.46,1.00) | |  |  | 0.72(0.48,1.07) |  |
|  | CSF | 1.35(1.00,1.81) |  |  | 1.26(0.94,1.70) |  | 1.56(0.83,2.96) |  |  | 1.48(0.78,2.78) |  | 0.78(0.37,1.64) |  |  | 0.76(0.36,1.61) |  | 1.28(0.85,1.94) | |  |  | 1.23(0.81,1.87) |  |
| MRI Lacunes | PET | 0.47(0.20,1.09) |  |  | 0.51(0.22,1.20) |  | 0(0,Inf) |  |  | 0(0,Inf) |  | 0.54(0.06,4.90) |  |  | 0.53(0.07,4.09) |  | 0.44(0.16,1.20) | |  |  | 0.47(0.17,1.33) |  |
|  | CSF | 2.42(1.01,5.78) | * |  | 2.03(0.85,4.86) |  | 12.35(1.06,143.8) | * |  | 7.61(0.69,84.47) |  | 0.47(0.05,4.24) |  |  | 0.58(0.07,4.96) |  | 2.28(0.73,7.12) | |  |  | 1.89(0.60,5.95) |  |
| MRI  microbleeds | PET | 2.08(1.07,4.03) | * |  | 1.75(0.90,3.41) |  | 2.15(0.60,7.67) |  |  | 1.94(0.55,6.86) |  | 1.62(0.43,6.12) |  |  | 1.30(0.36,4.68) |  | 2.24(0.82,6.12) | |  |  | 1.87(0.69,5.09) |  |
|  | CSF | 0.89(0.46,1.73) |  |  | 0.88(0.46,1.71) |  | 1.01(0.29,3.57) |  |  | 0.91(0.26,3.17) |  | 0.75(0.20,2.81) |  |  | 0.81(0.22,2.94) |  | 1.08(0.38,3.09) | |  |  | 1.04(0.38,2.88) |  |

*** - p<0.001, ** - p<0.01, * - p<0.05. P-values indicate the significance of the patient feature in the model. Uncorrected p-values and corrected p-values are reported per model, additionally corrected p-values for imputed data. False discovery rate (FDR) correction was performed for multiple comparisons. Cognitive scores have been multiplied by -1, therefore lower scores usually indicate higher odds ratios for amyloid positivity.
